# 3D Local Feature Learning and Analysis on Point Cloud Parts via Momentum Contrast

**DOI:** 10.3390/s26031007

**Published:** 2026-02-03

**Authors:** Xuanmeng Sha, Tomohiro Mashita, Naoya Chiba, Liyun Zhang

**Affiliations:** 1 Graduate School of Information Science and Technology, The University of Osaka, Suita, Osaka 565-0871, Japan; u418576a@ecs.osaka-u.ac.jp (X.S.); chiba@nchiba.net (N.C.); 2 Department of Engineering Informatics, Osaka Electro-Communication University, Neyagawa, Osaka 572-8530, Japan; 3 Graduate School of Frontier Sciences, The University of Tokyo, Kashiwa-shi, Chiba 277-8561, Japan; liyun.zhang@lab.ime.cmc.osaka-u.ac.jp

**Keywords:** 3D point cloud, self-supervised learning, contrastive learning, momentum encoder, local feature representation

## Abstract

Self-supervised contrastive learning has demonstrated remarkable effectiveness in learning visual representations without labeled data, yet its application to 3D local feature learning from point clouds remains underexplored. Existing methods predominantly focus on complete object shapes, neglecting the critical challenge of recognizing partial observations commonly encountered in real-world 3D perception. We propose a momentum contrastive learning framework specifically designed to learn discriminative local features from randomly sampled point cloud regions. By adapting the MoCo architecture with PointNet++ as the feature backbone, our method treats local parts of point cloud as fundamental contrastive learning units, combined with carefully designed augmentation strategies including random dropout and translation. Experiments on ShapeNet demonstrate that our approach effectively learns transferable local features and the empirical observation that approximately 30% object local part represents a practical threshold for effective learning when simulating real-world occlusion scenarios, and achieves comparable downstream classification accuracy while reducing training time by 16%.

## 1. Introduction

In recent years, 3D computer vision has advanced significantly alongside the development of 2D vision tasks, enabling essential applications including 3D object detection, classification, semantic segmentation, and reconstruction. However, unlike structured and continuous 2D images, 3D data presents unique challenges stemming from its inherent properties—unordered point sets, sparse spatial distributions, and irregular structures. These characteristics lead to substantial computational costs, labor-intensive annotation requirements, and inevitable information loss during data acquisition.

**Figure 1:**
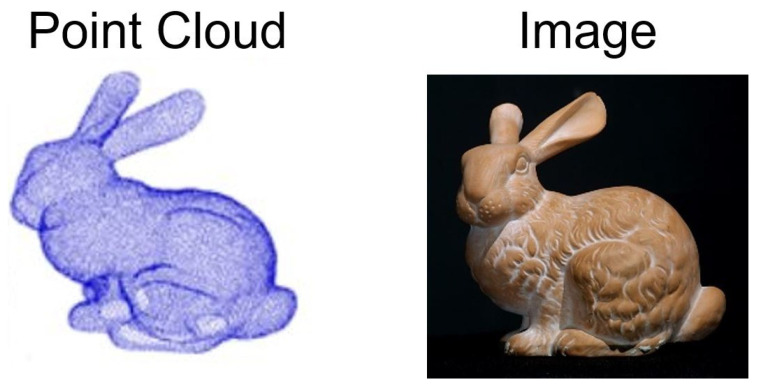
Samples of Point Cloud and Image [15].

Due to the exclusive properties of 3D data, different types of data structure are used for solving 3D vision tasks [1,2,3,4,5,6,7,8,9]. Point clouds represent the most fundamental format for capturing real-world 3D geometry. As shown in Figure 1, a point cloud is simply a collection of 3D coordinates in Euclidean space, where each point may contain additional attributes such as color, intensity, reflectivity, or normal information [10]. This unstructured nature, while preserving geometric fidelity, introduces significant obstacles for traditional supervised learning approaches [11,12,13,14]. The high dimensionality of point cloud data, combined with the scarcity of large-scale labeled datasets, makes it impractical to rely solely on supervised training mechanisms for extracting meaningful representations.

Self-supervised learning (SSL) has emerged as a promising solution, achieving remarkable success in natural language processing [16,17,18] and 2D vision tasks [19,20,21]. By learning from unlabeled data through carefully designed pretext tasks, SSL methods can extract useful representations without human annotations. Contrastive learning, a particularly effective SSL paradigm, encourages different augmented views of the same input to produce similar representations while separating representations from different inputs. For 3D point clouds, this approach leverages various data augmentation techniques to create multiple views, enabling the model to learn robust features through self-supervision [22]. This paradigm not only reduces computational costs [23] but also circumvents the expensive annotation process by generating pseudo-labels through clustering methods such as memory bank [19], while preventing information loss through well-designed contrastive objectives [24].

Despite these advances, existing 3D self-supervised contrastive learning methods predominantly focus on learning global shape representations of complete objects [25,26]. This global-only approach overlooks a critical aspect of real-world 3D perception: objects are frequently observed as partial views due to occlusion, sensor limitations, or viewpoint constraints. Recent study on 3D classification and segmentation have shown that model performance significantly degrades by 30–60% in accuracy when tested on partially visible or occluded objects, even when trained on complete shapes [27]. To address the importance of local information, some recent works focus on 3D local parts, defined as spatially contiguous subsets of points sampled from complete point clouds. DHGCN [28] voxelizes point clouds into parts to discover their contextual relationships, while LAA [29] introduces a local–global attention module that strengthens local cues through hierarchical design. More recently, Point-MAE [30] applies masked autoencoding to learn point cloud representations, and Point-CMAE [31] combines contrastive learning with masked autoencoding to improve feature learning. However, as shown in Table 1, these methods suffer from two fundamental limitations: they either lack robustness to partial observations by not learning directly from geometric local parts (Point-MAE, LAA, DHGCN), or they lack effective architectural and learning design by missing hierarchical structure or contrastive loss mechanisms (Point-MAE, DHGCN, Point-CMAE). None of these methods treat randomly sampled local parts as the primary training unit in contrastive learning, limiting their ability to learn discriminative features essential for recognizing objects under realistic partial observation conditions.

To address these gaps, we propose a modified momentum contrastive learning framework specifically designed for learning 3D local feature representations. As shown in Table 1, our method combines all critical capabilities lacking in previous approaches: learning from geometric local parts, focusing on occlusion scenarios, employing hierarchical architecture, and utilizing contrastive loss. Building upon the Momentum Contrast (MoCo) architecture [32], which introduced momentum-based contrastive mechanisms for 2D tasks, we adapt this structure for 3D point cloud processing. Unlike previous methods that rely on predefined part groupings or focus only on global shapes, our approach treats randomly sampled local parts as the fundamental training units in contrastive learning. We employ PointNet++ as the feature learning backbone to capture hierarchical local information and generate point cloud clusters as local parts through random local clustering combined with carefully designed 3D data augmentation strategies. By randomly sampling and learning from local parts of 3D objects, our framework explicitly models the partial observation scenarios commonly encountered in real-world 3D scenes, thereby learning robust and transferable local features that directly capture geometric information from local parts without requiring predefined part structures.

We conduct comprehensive experiments on ShapeNet [33,34] to evaluate our proposed method. Our investigation focuses on three key aspects: (1) the relationship between the size of local parts and feature learning capability, (2) the effectiveness of different data augmentation methods for local feature training, and (3) the transferability of learned features to downstream 3D object classification tasks. This work makes the following contributions:

- We present a momentum contrastive learning framework that addresses both robustness to partial observations and architectural design limitations by treating randomly sampled geometric local parts as the primary training unit with hierarchical feature extraction and contrastive loss.
- We systematically analyze how local parts’ size and data augmentation strategies affect feature learning, empirically observing that approximately 30% local parts represents a practical threshold for effective learning under controlled occlusion simulation, with random dropout and translation augmentation proving essential.
- We demonstrate that our pretrained models achieve comparable accuracy to models trained from scratch while reducing training time by 16%, validating the effectiveness of learned local features for downstream tasks.

## 2. Related Work

### 2.1. Self-Supervised Training

Self-supervised training has achieved remarkable success in natural language processing tasks such as GPT [16,18] and BERT [17], and has increasingly been adopted for vision tasks. Early self-supervised approaches for images imposed simple variations on inputs and extracted features by recovering the original data. These include semantic-label-based methods [35,36] and cross-modal-based methods [37,38,39]. However, these methods incur extra computational costs and memory overhead for generating labels and learning from other modalities.

Recently, contrastive learning has gained significant attention due to its ability to rely solely on the dataset itself by generating positive and negative samples through data augmentation. Wu et al. store previous feature encodings in a memory bank [19], while Momentum Contrast (MoCo) [32] extends this approach with an updating queue for negative sample features and a momentum update encoder, surpassing ImageNet-supervised counterparts in multiple detection and segmentation tasks. Other advances [20,21] have further demonstrated that self-supervised training is highly effective for 2D vision tasks.

For 3D vision tasks, Xie et al. proposed PointContrast [25], which augments 3D scenes with rotations and color transformations before contrasting transformed point clouds using a contrastive loss function. To address the limitation of requiring multiple views, Zhang et al. presented DepthContrast [26], which learns representations from depth maps using only single-view point cloud data. It constructs two augmented versions through data augmentation, with format-specific encoders generating spatial features from either point or voxel representations, ultimately obtaining global features for instance discrimination.

However, these works focus on pretraining with complete object shapes, thereby neglecting local part information. Recent studies have begun to address this limitation through different approaches. DHGCN [28] voxelizes point clouds into parts and predicts hop distances between parts to learn their contextual relationships, improving structural reasoning across point parts. LAA [29] introduces a local–global attention module that strengthens local cues while maintaining global context in transformer-based pretraining. Point-MAE [30] applies masked autoencoding to learn representations from incomplete point clouds, while Point-CMAE [31] extends this by incorporating contrastive learning to improve feature discrimination under occlusion scenarios. Work in other visual domains [40] has similarly shown benefits from structured region perception. Despite these efforts, existing methods remain limited. Firstly, most methods do not learn directly from randomly sampled geometric local parts, instead relying on predefined part grouping (DHGCN, LAA) or masking strategies (Point-MAE, Point-CMAE); secondly, they lack comprehensive design that combines hierarchical feature extraction with contrastive learning on local parts. Moreover, these methods do not systematically examine how the size and variation of local parts affect learned features, which is critical for understanding their effectiveness in handling partial observations commonly encountered in real-world 3D perception [28,29,41]. While diffusion-based approaches have shown promise in modeling motion trajectories for sequential tasks [42], their application to self-supervised point cloud feature learning remains unexplored.

Our work takes a different approach that addresses these limitations by focusing on the geometric local parts under real-world occlusion and the hierarchical contrastive learning design. We sample local parts directly as contrastive learning inputs and systematically investigate how local part characteristics affect feature representation. This provides a simple and direct method to learn geometric local parts without relying on handcrafted part definitions or complex architectural modifications, while maintaining the ability to handle partial observations through momentum-based contrastive learning on randomly sampled local parts.

### 2.2. Point Cloud Encoder

Point cloud encoders have emerged as essential components in 3D vision tasks due to the unique challenges of processing unstructured 3D data. Point-based models capture representations at the point-wise level. PointNet [43] pioneered the direct processing of raw point clouds by learning spatial encodings of individual points and aggregating them into a global signature. However, it suffers from the loss of local geometric information. To address this limitation, PointNet++ [44] introduced a hierarchical structure that extracts features while considering both local and global information through multiple stages of sampling and grouping. This hierarchical design with explicit local neighborhood aggregation makes it particularly well-suited for processing local point patches, as it can effectively capture fine-grained geometric patterns within localized regions.

Building upon PointNet++, subsequent works have proposed various enhancements. Qian et al. introduced PointNeXt [45], which incorporates a separable MLP and an inverted residual bottleneck design. Hu et al. proposed RandLA-Net [46], which employs random point sampling and increases the receptive field through an efficient local feature aggregation module. Ma et al. presented PointMLP [47], a residual model that applies a geometric affine module instead of a local geometry extractor.

Since the introduction of the Transformer [48], many encoders have adopted this architecture for point cloud processing. Guo et al. proposed Point Cloud Transformer (PCT) [49], which provides permutation invariance and brings the transformer to point cloud feature learning. Pang et al. developed Point-MAE [30], a masked autoencoder that addresses local information leakage.

While these works propose point cloud encoders primarily for supervised training, our work leverages the point cloud encoder within a self-supervised training framework specifically designed for extracting local features. To efficiently represent 3D local parts, we adopt PointNet++ as our feature learning backbone due to its hierarchical structure and effective local neighborhood capturing capabilities, which are particularly advantageous for processing local point patches. Furthermore, the learned features can be directly transferred to downstream tasks such as 3D object classification and detection.

## 3. Method

To learn discriminative 3D local features through contrastive learning, we propose a modified momentum contrastive pretraining architecture specifically designed for local point cloud features. This section presents the overall framework architecture, including the momentum contrastive learning mechanism with its contrastive loss and momentum update strategy, the point cloud feature learning backbone, and the data processing pipeline comprising augmentation methods and local cluster sampling. In this framework, the sampled local clusters are presented as the geometric local parts of point cloud.

### 3.1. Momentum Contrastive Learning Structure

**Figure 2:**
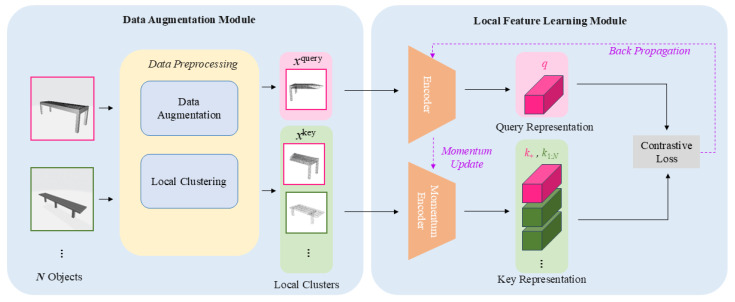
Momentum Contrastive Learning Structure for 3D Local Features. Solid arrows denote the data flow, while dashed arrows represent parameter updates (back-propagation and momentum update). Pink and green colors distinguish the query and key branches, respectively.

The proposed framework is illustrated in Figure 2. The architecture consists of two primary modules: a data augmentation module and a local feature learning module. The data augmentation module processes input 3D point clouds into transformed local clusters that serve as queries and keys. The local feature learning module then learns discriminative local features from these query and key clusters through contrastive learning.

The complete data flow proceeds as follows: First, an input point cloud undergoes data augmentation transformations. Second, the KD-Tree algorithm samples local clusters from the augmented point cloud. Third, these local clusters are processed by the query encoder and momentum encoder to generate feature representations. Finally, the InfoNCE loss is computed to train the encoders. This step-by-step pipeline ensures that geometric local information is effectively captured and learned through contrastive objectives.

Data Augmentation Module: The data augmentation module takes a complete point cloud as input and outputs a batch of transformed local clusters as local parts. Within each batch, the local clusters are designated as queries and keys. A query–key pair is considered positive if both clusters originate from the same CAD model; otherwise, they form negative pairs. The data augmentation methods generate local clusters with diverse geometric variations, enabling the momentum contrast framework to learn robust local features. The local clustering method randomly samples local parts from the augmented point clouds, producing local clusters that capture different geometric patterns.

Local Feature Learning Module: This module learns local features from query and key clusters while updating the encoders through momentum contrast. To ensure feature consistency during contrastive learning, both the query encoder and momentum encoder share the same architectural structure. During training, the query encoder is updated via standard back-propagation, while the momentum encoder is updated through a momentum mechanism, resulting in models with identical architecture but different parameters. The contrastive loss function encourages the query features to match their corresponding positive key features while remaining distinct from negative key features, thereby training the encoders to learn discriminative local features.

#### 3.1.1. Contrastive Loss

Local clusters are encoded into query and key features by the query encoder and momentum encoder, respectively. Given a batch size of *N* samples, the encoder produces one query feature *q* and a set of key features {$k_{+},k_{1},k_{2},\ldots ,k_{N-1}$}. These key features constitute a dictionary for contrastive learning. The positive pair $\left(q,k_{+}\right)$ for i=1,2,…,N−1 come from different point clouds. To encourage the query feature *q* to be similar to its positive key $k_{+}$ and dissimilar to negative keys, we employ the InfoNCE loss [50]:

(1) $$L_{q}=-\log\frac{exp(q\cdot k_{+}/\tau )}{exp\left(q\cdot k_{+}/\tau \right)+\sum_{i=1}^{N-1}exp\left(q\cdot k_{i}/\tau \right)},$$

where τ is a temperature hyperparameter that controls the smoothness of the softmax distribution. We set τ=0.07 following standard practice in contrastive learning. By minimizing this loss, the model learns to pull together local features from the same point cloud in the latent space while pushing apart features from different point clouds. This unsupervised objective enables the encoders to capture informative geometric local information through contrastive learning on local parts of point cloud.

#### 3.1.2. Momentum Update

The contrastive loss trains the encoders to produce discriminative local features for both query and key clusters. The dictionary of key features is maintained as a queue that is progressively updated: as each new mini-batch is processed, its key features are enqueued while the oldest mini-batch is dequeued. This queue mechanism maintains a large and relatively current set of negative samples for contrastive learning. We set the queue size to 65,536 to provide sufficient negative samples for effective contrastive learning.

However, applying standard back-propagation to update both encoders would cause inconsistencies in the key representations stored in the queue. To address this issue, we employ a momentum update mechanism for the momentum encoder:

(2) $$\theta_{k}⟵m\theta_{k}+\left(1-m\right)\theta_{q},$$

where $\theta_{q}$ and $\theta_{k}$ denote the parameters of the query encoder $f_{q}$ and momentum encoder $f_{k}$, and m=0.999 is the momentum coefficient following MoCo [32]. This ensures $f_{k}$ evolves slowly, maintaining consistency of key features in the queue despite being generated at different training iterations.

### 3.2. Point Cloud Feature Learning Backbone

To effectively learn informative local features from point clouds, we adopt PointNet++ [44] as our feature learning backbone. PointNet++ is particularly well-suited for local feature learning due to its hierarchical structure that explicitly captures multi-scale local information through successive sampling and grouping operations. This design enables the network to aggregate information from local parts at multiple scales, making it ideal for extracting discriminative features from them.

The network takes XYZ coordinates of 3D points as input and employs a U-Net-like architecture [51]. In our implementation, each local parts contains 1024 points, represented as a 1024×3 matrix of XYZ coordinates of local clusters. The hierarchical sampling and grouping operations progressively reduce the point set size while increasing the feature dimensionality, allowing the network to capture both fine-grained local details and broader contextual information. Finally, the per-point features are then max-pooled to obtain a global feature vector for the local parts, which serves as the input to the projection head for contrastive learning.

The explicit local-to-global hierarchical structure of PointNet++ naturally aligns with our objective of learning geometric local features. By processing local parts through this backbone, the network learns to identify distinctive local information while maintaining awareness of the broader spatial context within each cluster.

### 3.3. Data Processing

#### 3.3.1. Data Random Augmentation Methods

To improve the robustness of learned local features and enable the model to handle various real-world scenarios, we apply three standard 3D augmentation methods: random input dropout, random rotation, and random translation. These augmentations generate diverse views of local parts, encouraging the model to learn invariant geometric local features.

Random input dropout randomly removes 20–30% of points from each local parts, simulating the partial occlusions and missing data frequently encountered in real-world 3D sensing. This augmentation forces the model to recognize local information even when parts of the structure are absent.

Random rotation applies random rotations around the vertical axis (z-axis) within the range of 0–360°, generating local parts with different orientations. This augmentation ensures that learned features are invariant to object orientation, which is crucial for recognizing local parts regardless of viewpoint.

Random translation randomly shifts the local parts positions within a range of ±0.2 m along each axis, simulating viewpoint variations during 3D data acquisition. This augmentation encourages the model to focus on relative geometric relationships within local parts rather than absolute spatial positions.

These augmentation methods work together to create a rich variety of local parts appearances from each input point cloud, enabling the momentum contrastive framework to learn robust and generalizable geometric local features. We conduct ablation studies to evaluate the individual contribution of each augmentation method to the overall performance.

#### 3.3.2. Local Clustering Method

To sample local clusters as local parts from complete point clouds, we employ the K-dimensional Tree (KD-Tree) algorithm, which is a space-partitioning data structure that efficiently organizes k-dimensional points in a tree structure for fast range searches and nearest neighbor queries. The KD-Tree algorithm is particularly suitable for local cluster sampling due to its computational efficiency, which significantly accelerates the pretraining process.

**Figure 3:**
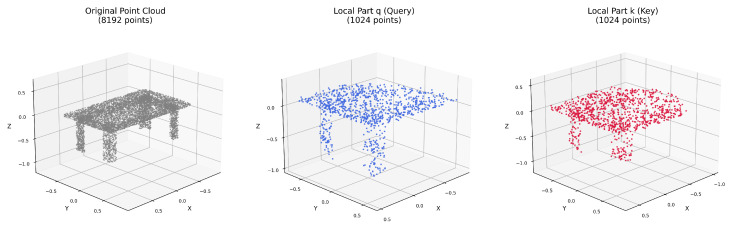
Example of Query and Key Local Parts after Local Clustering Method.

The local clustering process proceeds as follows: First, we randomly select a center point from the augmented point cloud. Second, we use the KD-Tree to identify the *K* nearest neighbors of the center point, where *K* is set to 1024 to form a complete local cluster. This approach ensures that each local cluster captures a coherent local part while the random center selection provides diverse local samples across the entire point cloud. The Figure 3 illustrates the sampled query and key local parts from the original point cloud after local clustering method. By sampling local clusters in this manner, we can generate multiple views of local parts from a single point cloud, each representing different geometric patterns and structural characteristics. This random sampling strategy enables the model to learn from varied geometric local part configurations, enhancing its ability to recognize partial shapes and local information in downstream tasks.

## 4. Experiments

We conduct two stages of experiments to evaluate our proposed momentum contrastive pretraining framework for 3D local features. The first stage investigates pretraining effectiveness by examining how local cluster size and data augmentation methods influence the learning of local feature. The second stage evaluates the transferability of learned features through downstream 3D object classification tasks, comparing models with and without pretraining. Through these experiments, we demonstrate that our method effectively learns discriminative local features that accelerate downstream task training.

### 4.1. Dataset

**Figure 4:**
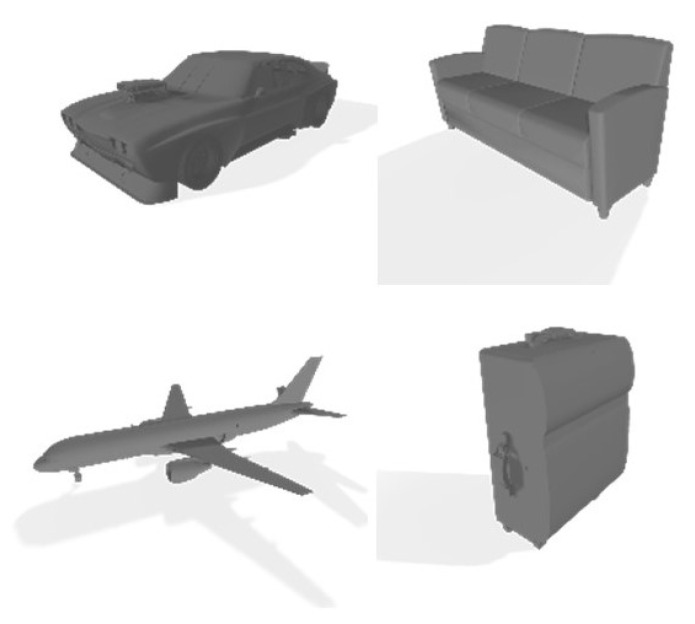
Samples of ShapeNet.

We use ShapeNet [34] as our pretraining dataset. As illustrated in Figure 4, it is a collection of single-object CAD models containing 57,448 objects across 55 categories, developed by researchers from Stanford University, Princeton University, and the Toyota Technological Institute at Chicago. This dataset is widely adopted for 3D vision tasks and provides rich geometric information for computer graphics and vision research.

For pretraining experiments, we randomly select 30% of the ShapeNet dataset as our training set. This subset size is chosen to accelerate the pretraining process while maintaining sufficient diversity to validate our method’s effectiveness across different geometric local parts. Our primary objective is to demonstrate the viability of local feature learning through momentum contrastive pretraining rather than achieving maximum accuracy on the full dataset. For the downstream classification task, we select five representative object categories including table, chair, lamp, bench, and bookshelf. It focus the evaluation on how learned local features influence classification performance without introducing complexity from large-scale multi-class scenarios. This focused downstream setting allows us to isolate the effect of local feature pretraining on transfer learning efficiency.

Each local cluster is sampled to contain 1024 points from the input point cloud. We set the batch size to 64 and the learning rate to 0.007 for pretraining. All pretraining experiments are conducted for 200 epochs to ensure convergence. All experiments were conducted on a workstation equipped with four NVIDIA Tesla V100-PCIE GPUs (32 GB, NVIDIA Corporation, Santa Clara, CA, USA). The software environment consisted of Python (v3.7.10), PyTorch (v1.9), and CUDA (v12.0). These computational resources supported the pretraining and analysis of 3D local feature.

### 4.2. Momentum Contrast Pretraining

We design two comparison experiments to systematically evaluate our momentum contrast pretraining method. The first experiment examines the relationship between local cluster size and feature learning capability, aiming to identify the minimum cluster size required for effective contrastive learning. The second experiment investigates the contribution of different data augmentation methods to local feature extraction.

#### 4.2.1. Training with Different Partial Scales

**Figure 5:**
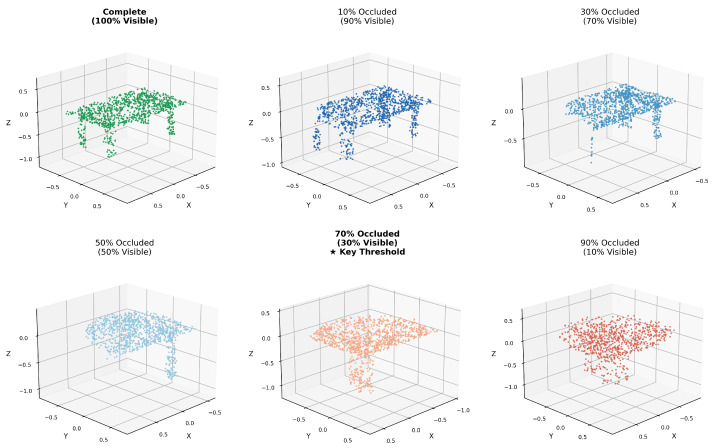
Visual Comparison of Different Local Part Sizes from 100% to 10%. The star (★) indicates the 30% visibility threshold identified as the minimum requirement for effective learning.

This experiment evaluates how local cluster size affects the learning of local features through momentum contrast. It is important to note that our findings are constrained by the specific experimental configuration: point cloud density of 1024 points and PointNet++ as the feature backbone. We train models with local clusters ranging from 20% to 90% of the complete object, maintaining identical data augmentation strategies across all settings. This setup is designed to simulate real-world occlusion scenarios where objects are frequently observed as partial views. The results enable us to determine whether momentum contrast is effective for learning 3D local features and to characterize the relationship between cluster size and feature learning capability under these specific conditions. Figure 5 visualizes the different scales of local parts, ranging from complete objects (100% visible) to severely occluded observations (10% visible).

The results are presented in Table 2. We report two metrics: Acc@1 (top-1 classification accuracy, where the highest probability classification corresponds to the positive key) and Acc@5 (top-5 classification accuracy, where the positive key appears among the top five predictions). As shown in Table 2, both Acc@1 and Acc@5 increase as local cluster size grows, confirming that our contrastive learning framework can effectively learn from local features. Notably, clusters containing 33% to 90% of the object achieve similar performance levels (Acc@1 ranging from 44.71% to 51.68%), indicating that moderate-sized local parts contain sufficient geometric information for contrastive learning.

However, performance degrades dramatically when cluster size falls below 30%, with Acc@1 dropping to 25.62% at 30% and nearly 0% at 20% and 25% cluster size. This sharp decline suggests that clusters smaller than 30% lack sufficient geometric local information to form stable positive pair relationships in the contrastive learning framework. When local parts become too small, they may represent ambiguous or incomplete geometric patterns that cannot be reliably distinguished from negative samples, causing the contrastive learning objective to fail. Based on these empirical observations from ShapeNet with 1024-point sampling, we observe that 30% represents the threshold for maintaining effective learning under our specific configuration, serving as the minimum identifiable partial observation ratio capable of supporting semantic discrimination. This finding demonstrates that our framework can learn from partial observations that simulate real-world occlusion scenarios, where approximately one-third of an object remains visible.

#### 4.2.2. Training with Different Data Augmentation Methods

This experiment investigates the contribution of each data augmentation method to local feature learning by systematically removing individual augmentation techniques. We train models with local clusters containing 50% of the complete object and compare performance across different augmentation combinations. The results are shown in Table 3.

The results reveal that random input dropout and random translation are essential for effective local feature learning, while random rotation exhibits minimal impact within our specific experimental configuration. When random input dropout is removed, performance collapses to near-zero (Acc@1 = 0.59%), indicating that this augmentation is critical for learning robust local features. This dramatic degradation suggests that dropout forces the model to recognize geometric local information even when parts of the structure are missing, simulating the partial occlusions encountered in real-world scenarios. Without this augmentation, the model may overfit to complete local clusters and fail to generalize to partial views.

Similarly, removing random translation significantly reduces performance. This finding suggests that translation augmentation helps the model focus on relative geometric relationships within local parts rather than absolute spatial positions, encouraging the learning of position-invariant local features. In contrast, removing random rotation has negligible impact on performance in our experiments. This observation can be attributed to two factors specific to our experimental setup: first, local patches at the scale of 50% often exhibit nearly isotropic geometric patterns where rotational variations introduce minimal distinguishable differences; second, ShapeNet models are typically pre-aligned with consistent up-vectors, reducing the natural rotational diversity that rotation augmentation aims to capture. These ablation results confirm that our augmentation strategy is well-designed, with dropout and translation playing complementary roles in promoting robust local feature learning.

### 4.3. Downstream Tasks

To further validate that our momentum contrastive pretraining method effectively learns transferable local features, we conduct downstream 3D object classification experiments comparing models with and without pretraining. Before reporting quantitative classification results, we first analyze the structure of the learned local feature space using feature visualization. This analysis provides qualitative insight into how local parts from different object categories are organized in the embedding space and helps interpret the behavior of the pretrained features prior to downstream evaluation. Then we select classification as our downstream task to establish a proof-of-concept that directly tests whether the pretrained encoder produces semantically discriminative feature representations when trained on partial observations. This task allows us to isolate and measure the transfer learning efficiency gained from our local feature pretraining approach without introducing the complexity of dense prediction tasks. The pretrained model is obtained using the optimal configuration identified in the pretraining experiments: local parts containing 50% of the object and all three augmentation methods. Following standard protocols [44], we maintain the same dataset settings and data processing pipeline as the pretraining stage. For downstream training, we use 200 epochs with a learning rate of 0.001, a batch size of 24, and the Adam optimizer.

#### 4.3.1. Feature Space Analysis of Learned Local Parts

**Figure 6:**
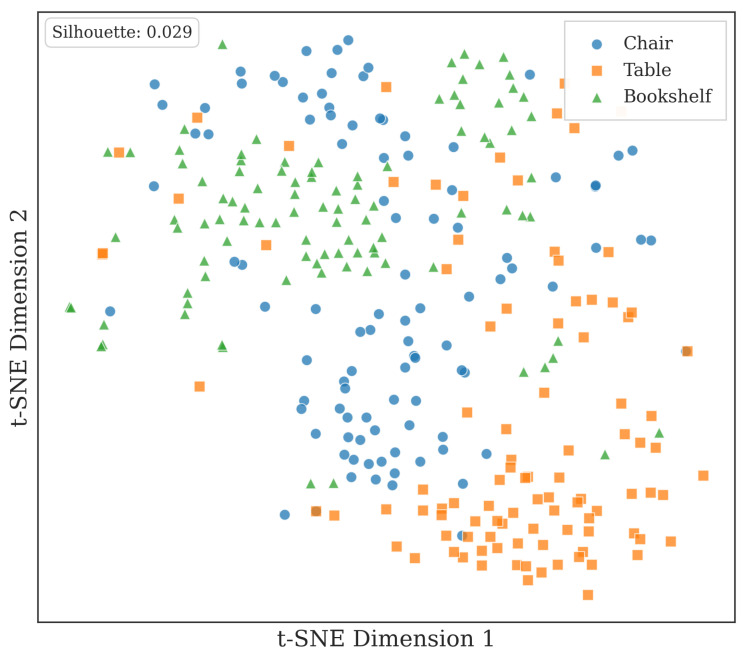
t-SNE visualization of learned local part features from chair, table, and bookshelf categories.

To provide qualitative insight into the learned feature representations, we visualize the local part feature space using t-SNE. As shown in Figure 6, local parts from chair, table, and bookshelf categories form coherent and partially separated distributions in the embedding space. Each category occupies a consistent region, while geometrically similar local structures exhibit close proximity across categories. This indicates that the learned features capture stable and transferable geometric patterns from local parts, supporting effective discrimination at the part level while maintaining generalization across object categories.

#### 4.3.2. 3D Object Classification on Local Parts

We first train the classification model using our proposed momentum contrast pretraining method on five selected ShapeNet categories. The pretraining achieves a best top-1 accuracy of 51.42% at epoch 132, with the entire pretraining process requiring approximately 6.5 h.

We then conduct two comparison experiments for 3D local parts classification. The first baseline trains a randomly initialized PointNet++ classification model to classify local parts from the five ShapeNet categories. The second experiment fine-tunes the pretrained PointNet++ model obtained from the best momentum contrast pretraining run. The classification results are presented in Table 4.

After 200 epochs of downstream training, both models achieve comparable final performance. However, the pretrained model reaches its best performance significantly faster, achieving optimal results at epoch 168 with a total training time of 3 h and 38 min, compared to epoch 199 and 4 h and 19 min for the baseline model. This represents approximately 16% reduction in training time to reach comparable accuracy.

The accelerated convergence with pretraining can be attributed to the initialization provided by momentum contrastive learning. During pretraining, the model learns to organize local features into a structured latent space where geometrically similar local parts are pulled together while dissimilar regions are pushed apart. This pretraining phase establishes a meaningful initial feature representation that captures fundamental geometric local information. When fine-tuned for downstream classification, the classification head can leverage this structured feature space, enabling faster convergence compared to learning from random initialization. These results demonstrate that our momentum contrastive pretraining method successfully learns transferable local features from 3D point clouds, accelerating downstream training while maintaining competitive final accuracy. The comparable final performance indicates that the pretrained features do not sacrifice representational capacity, while the reduced training time validates the practical utility of local feature pretraining for 3D understanding tasks.

## 5. Discussion

Our experimental results demonstrate that momentum contrastive learning can be successfully adapted to learn discriminative features from local parts of point clouds. This addresses a key limitation of existing self-supervised methods that focus exclusively on complete object shapes.

### 5.1. The 30% Threshold and Its Implications

Our empirical observation that approximately 30% object local part serves as a practical threshold for effective contrastive learning provides important insight into the information requirements for local feature learning. This threshold suggests that while geometric local information is crucial for object recognition, a certain minimum amount of structural context is necessary to form stable positive pair relationships in contrastive learning. This observation aligns with previous studies on 3D object recognition under occlusion, which have shown significant performance degradation when less than 30% of an object is visible.

It is important to clarify that this 30% threshold is specific to our experimental configuration (1024-point sampling, PointNet++ backbone, ShapeNet dataset) and represents a lower bound established under controlled conditions. We deliberately chose clean synthetic data to isolate the relationship between geometric information loss and feature learning without confounding effects from sensor noise or scanning artifacts. Three factors can significantly influence this ratio in practice: (1) point cloud density directly affects available geometric information—higher density may allow smaller percentages to remain effective, while sparser clouds may require larger proportions; (2) object complexity determines the minimum recognizable ratio, as simple uniform structures need larger proportions while complex objects with rich local details remain recognizable from smaller partial observations; (3) backbone architecture shapes the capacity to extract local information, and alternative architectures such as Transformers or graph neural networks may learn from smaller local parts by better capturing contextual relationships.

### 5.2. Data Augmentation Strategies

The effectiveness of random input dropout and random translation as augmentation methods, combined with the minimal contribution of random rotation, reveals important characteristics of geometric local feature learning. The critical role of dropout suggests that learning to recognize partial patterns is essential for robust local feature representations, as this augmentation directly simulates the occlusions and missing data encountered in real-world 3D sensing. The importance of translation augmentation indicates that position-invariant features are valuable for local pattern recognition. In contrast, the negligible impact of rotation suggests that at the scale of 50% local parts, geometric patterns exhibit inherently weak directional characteristics, and ShapeNet models are typically pre-aligned with consistent up-vectors. These findings differ from previous work on complete object recognition, where rotation augmentation typically provides substantial benefits, highlighting that local feature learning requires a distinct augmentation strategy.

### 5.3. Transferability to Real-World Scenarios

The transferability of features learned on synthetic ShapeNet data to real-world scenarios warrants careful consideration. Our augmentation strategies establish this bridge through two mechanisms. First, the 20–30% random dropout not only simulates occlusion but also replicates the “missing point” phenomenon inherent in LiDAR scanning, where surface reflectivity variations and grazing angles cause incomplete coverage. Second, the ±0.2 m random translation forces the model to learn relative geometric relationships rather than absolute positions, directly addressing the viewpoint variations in practical 3D sensing applications.

The framework’s focus on local geometric patterns makes it suitable for recognizing repetitive structures commonly found in large-scale environments. In architectural contexts such as building facades, where elements like windows, columns, or decorative moldings recur across the scene, the learned local features can identify these consistent geometric patterns without requiring global shape information. Individual window frames from various positions on a facade would produce similar feature representations despite appearing in different global contexts. The random sampling strategy during training naturally exposes the model to diverse instances of such repetitive elements, enabling generalization across multiple occurrences of the same local pattern.

### 5.4. Practical Deployment Considerations

The downstream classification results, showing comparable final accuracy but reduced training time with pretraining, demonstrate that momentum contrastive learning captures transferable geometric local information. While the 16% time reduction may appear modest, it validates the effectiveness of the structured latent space established through self-supervised learning and becomes valuable in systems where computational resources are constrained or rapid model adaptation is required.

The PointNet++ backbone employed in our framework incorporates multi-scale grouping operations that provide inherent resilience to point density variations through adaptive neighborhood aggregation. Regarding the 30% visibility threshold, this boundary carries practical significance: when less than 30% of an object remains visible, the geometric information typically becomes too fragmented to maintain semantic recognizability. This threshold represents a fundamental limitation imposed by information availability rather than a model-specific constraint. For realistic scenarios with sensor noise, variable density, and occlusion artifacts, this 30% boundary establishes a clear performance expectation, indicating when sensor fusion or multi-view integration becomes necessary.

Real-world scenarios with these additional complexities would likely require larger partial ratios for effective learning. Future work will validate these findings on real-world datasets such as ScanNet or ModelNet with realistic occlusion to establish practical thresholds that account for sensor noise and acquisition artifacts. Additional directions include qualitative visualization of learned features, extension to dense prediction tasks such as semantic segmentation, and investigation of transformer-based architectures that may better capture long-range dependencies while maintaining local geometric awareness.

## 6. Conclusions

This work proposed a momentum contrastive learning framework specifically designed for learning 3D local feature representations from point clouds, addressing the previously unexplored problem of self-supervised learning directly from partial point cloud observations. Building upon the MoCo architecture, we adapted the momentum-based contrastive mechanism to process randomly sampled local clusters and designed a data processing pipeline combining random input dropout, random translation, and local KD-Tree sampling to generate diverse local clusters that simulate real-world partial observation scenarios. Systematic experiments on ShapeNet demonstrated that, under our specific experimental configuration with 1024-point sampling and PointNet++ backbone, approximately 30% object local part represents an empirical threshold for effective contrastive training when simulating real-world occlusion scenarios, with random input dropout and random translation proving essential for learning robust local features. Downstream classification experiments validated that models pretrained with our approach achieve comparable final accuracy to models trained from scratch while reducing training time by approximately 16%, confirming the effectiveness and transferability of our learned local features. This work demonstrates that momentum contrastive learning can be successfully adapted to learn discriminative features from local parts of point cloud, providing a simple and effective approach for geometric local feature learning that benefits downstream 3D understanding tasks through improved training efficiency.

## Figures and Tables

**Table 1:** Comparative Analysis of Local Awareness Capabilities. ✓ and × indicate the presence and absence of a feature, respectively.

Method	Robustness to Partial Observations		Architectural & Learning Design		
	**Geometric Local Parts**	**Occlusion Focus**	**Hierarchical**	**Contrastive Loss**	**Augmentation**
Point-MAE [30]	×	×	×	×	Masking
LAA [29]	×	×	✓	×	Attention
DHGCN [28]	×	×	✓	×	Voxelization
Point-CMAE [31]	×	✓	×	✓	Masking
Ours	✓	✓	✓	✓	Dropout & Translation

**Table 2:** Sensitivity Analysis of Cluster Scale on Local Feature Learning.

Percentage of Local Cluster Size	Loss	Acc@1 [%]	Acc@5 [%]
90%	8.05	44.71	50.57
80%	7.90	50.83	51.06
66%	8.03	51.68	51.68
50%	8.13	50.65	51.03
33%	8.00	50.59	50.62
30%	8.03	25.62	25.98
25%	8.52	0.31	1.24
20%	8.21	0.28	1.08

**Table 3:** Comparison Result with Different Data Augmentation Methods.

Data Augmentation Methods	Loss	Acc@1 [%]	Acc@5 [%]
All	8.13	50.65	51.03
w/o random input dropout	8.34	0.59	1.45
w/o random rotation	8.19	49.85	50.98
w/o random translation	8.36	11.29	12.37

**Table 4:** Comparison Result of 3D Object Classification on Local Parts.

	w/ Pretrained Model	w/o Pretrained Model
Best Instance Accuracy [%]	81.35	81.57
Best Class Accuracy [%]	75.70	76.08
Training Time Cost	3 h 38 m	4 h 19 m
Epoch Number for Best Result	EP168	EP199

## Data Availability

The data presented in this study are available from the corresponding author upon reasonable request.
